# Artificial intelligence for surgical management of benign esophageal disease: scoping review and evidence mapping

**DOI:** 10.1007/s00423-026-04050-1

**Published:** 2026-04-20

**Authors:** Alberto Aiolfi, Quan Wang, Pietro Mascagni, Davide Bona, Nicola Leone, Luigi Bonavina

**Affiliations:** 1 Department of Biomedical Sciences for Health, I.R.C.C.S. Ospedale Galeazzi – Sant’Ambrogio, Division of General Surgery, University of Milan, Milan, Italy; 2 Department of Pharmacy, Health and Nutrition Sciences, Azienda Ospedaliera di Cosenza, Division of General and Foregut Surgery, University of Calabria (UNICAL), Rende, (Cosenza), Italy; 3 Ambulatory Surgery Center & Department of Hernia and Abdominal Wall Surgery & Surgical Center for Gastroesophageal Reflux Disease, Xijing Hospital, the Fourth Military Medical University, Xi’an, China; 4 Bioimage Analysis Center, Fondazione Policlinico Universitario Agostino Gemelli IRCCS, Rome, Italia; 5 Institute of Image-Guided Surgery,IHU-Strasbourg, Strasbourg, France; 6 Artificial Intelligence Laboratory, Department of Mathematics and Computer Science, University of Calabria (UNICAL), Rende, (Cosenza), Italy

**Keywords:** Esophagus, Artificial intelligence, Machine learning, Deep learning, Lower esophageal sphincter, Laparoscopic surgery, Robotic surgery

## Abstract

**Background:**

Artificial intelligence (AI) has seen considerable growth mainly in surgical oncology, with current applications primarily centered on cancer diagnosis, staging, treatment planning, and outcomes prediction. Aim of the present scoping review was to describe the actual evidence and future perspectives of AI-application in the field of benign esophageal diseases.

**Methods:**

This scoping review summarizes current evidence on AI utilization in the diagnosis and surgical management of esophageal benign disease such as achalasia, Barrett’s esophagus, gastroesophageal reflux disease (GERD), hiatus hernia (HH), and Zenker diverticulum. PubMed, Scopus, Web of Science, Cochrane Library, and Google Scholar databases were searched until November 2025.

**Results:**

Overall, 37 studies published were included. The integration of AI within the surgical protocols of tertiary referral centers may offer the potential to enhance multidisciplinary decision-making, provide intraoperative assistance, and lead to improved patient outcomes by personalizing treatment of reflux disease, motility disorders and esophageal diverticula. Also, there is an urgent need of responsible AI development and implementation to support surgical education through objective skill assessment, simulation-based training, and competency evaluation. Machine learning, deep learning and hybrid models are still underexplored. Since continuous learning and system adaptability are crucial in healthcare, collaborative efforts to develop robust and validated patient-centered AI tools that align with real-world surgical workflow have the potential to uncover hidden trends and to deliver reliable predictions. Ultimately, AI applications within esophageal surgery must adhere to the ethical standards that define surgical practice: safety, transparency, accountability, equity, and dedication to patient welfare. By ensuring that innovation remains aligned with these foundational principles, AI can serve to elevate both the precision of surgical care and the preparation of future surgeons.

**Conclusions:**

AI can improve every stage of surgical care for benign esophageal disease, from diagnosis to postoperative management. It may also help standardize surgeon training and speed up learning for laparoscopic and robotic procedures. Realizing AI’s full benefits will require strong research, ethical practices, and thorough surgeon education.

## Introduction

Despite efforts in multidisciplinary treatment, some aspects of esophageal disease remain controversial due to lack of expert consensus, heterogeneity of diagnostic and therapeutic approach, and variability of patients’ outcomes. Gastroesophageal reflux disease (GERD) is a chronic pathological condition leading to persistent symptoms and significant complications [1]. It affects up to 20% of the population in developed countries, with a major impact in quality of life and substantial healthcare costs [2]. Pharmacological treatment with proton pump inhibitors is the first-line approach, but surgical intervention may be warranted for patients with refractory symptoms, esophagitis or Barrett’s esophagus, and those presenting with hiatal hernia (HH) [3]. Improved understanding of the close relationship between the diaphragm and esophagogastric junction has recently enabled more tailored antireflux procedures and holds promise for improved clinical outcomes [4]. Also, esophageal motility disorders such as achalasia and Zenker diverticulum may potentially benefit from more and precise endoscopic procedures (peroral myotomy), but the criteria for patients’ selection remain highly debated [5, 6].

The rise and rapid spread of artificial intelligence (AI) technology can offer transformative benefits in the surgical therapy of esophageal disorders by leveraging advances in machine-learning (ML), deep learning, and computer vision [7]. Implementation of AI has the potential to augment decision-making and surgical planning, intraoperative navigation, robotic assistance, and postoperative monitoring, thus opening interesting scenarios in oncological research [8–13]. In contrast to malignant disease, where surgical management prioritizes oncologic clearance, procedures for benign esophageal disorders focus primarily on restoring physiological function and quality of life [14]. As a result, these conditions impose more stringent requirements for patient selection, procedure choice, and tailored surgical training [15]. Consequently, benign esophageal diseases present both unique opportunities and distinct challenges for AI integration in clinical practice, with the aim to improve objective and patient-reported outcomes while minimizing overtreatment and reducing recurrence rates [16].

The present study critically appraises current and emerging applications of AI in the management of benign esophageal disorders to identify knowledge gaps and to highlight trends and research priorities. Through the integration of insights from current research, our aim is to clarify the advantages, challenges, and possible developments of AI within this particular area of surgery.

## Methods

### Search strategy

A scoping review was conducted to map the utilization of AI on diagnosis and surgical management of benign esophageal diseases such as esophageal achalasia, Barrett’s esophagus, GERD, HH, and Zenker diverticulum. The review was performed in accordance with the Preferred Reporting Items for Systematic Reviews and Meta-Analyses Extension for Scoping Reviews (PRISMA-ScR) [17]. The literature search was conducted by three authors (AA, PM, QW) to identify all English-written published articles reporting data on the utilization of AI on the diagnosis and treatment of benign esophageal disease. PubMed, Scopus, Web of Science, Cochrane Library, and Google Scholar databases were consulted matching the terms “AI” and “ML” and “deep learning” and “benign esophageal disease” and “GERD” and “Barrett esophagus” and “hiatus hernia” and “dysphagia” and “achalasia” and “diverticulum” with “AND” or “OR” until 10th November 2025. The search was completed by consulting the listed references of each article. All the articles, case reports, and case series were included in this review while abstracts were excluded. The study was approved by the local Institutional Review Board (#0467–2025). Informed consent was not necessary for the literature review.

### Extracted data

The following variables were analyzed: author(s) and year of publication, country and study design, sample size, AI clinical application (diagnosis, prognosis, treatment monitoring, surveillance), key outcomes, findings, and limitations. Eligible esophageal benign diseases included: esophageal achalasia, Barrett’s Esophagus, GERD, HH, and Zenker diverticulum.

### Critical appraisal of individual sources of evidence

Data were extracted using a standardized data charting form developed prior to data extraction. The charting process was iterative, allowing refinement of extracted variables as familiarity with the literature increased. Data were independently charted by three authors (AA, PM, QW), with discrepancies resolved by consensus. The study selection process was documented using a PRISMA flow diagram, detailing the number of records identified, screened, excluded, and included. Consistent with scoping review methodology, formal risk-of-bias assessment was not performed. However, methodological limitations and potential sources of bias were recorded and considered during interpretation of the findings. Extracted data were synthesized using a descriptive and narrative approach. A review protocol was developed prior to study initiation.

## Results

**Fig. 1:**
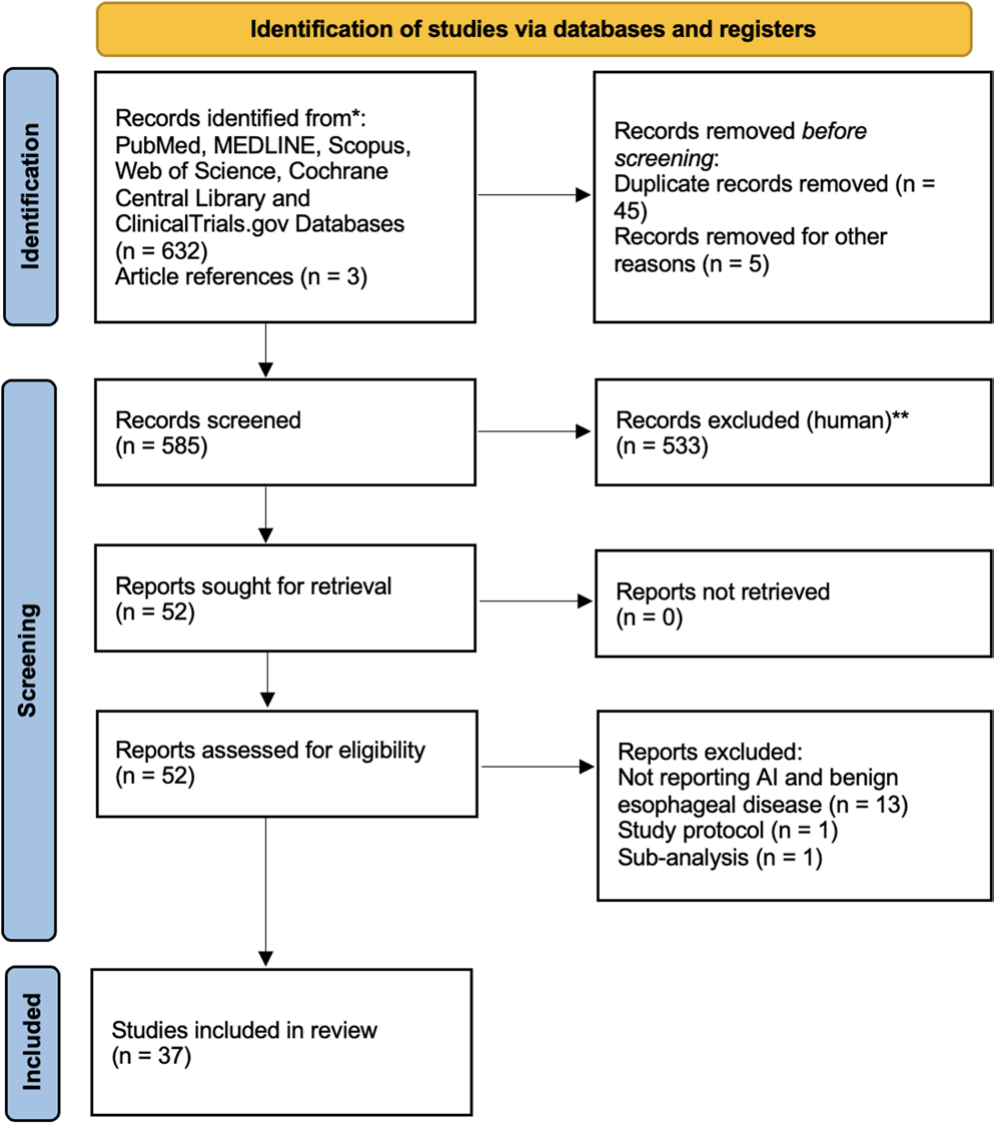
Preferred Reporting Items for Systematic Reviews and Meta-Analysis (PRISMA) flow diagram of study selection

A total of 37 studies were included in this review [18–54] (Fig. 1). Each publication presented findings related to the application of AI in the diagnosis and management of benign esophageal disease. Due to variability in reporting methods and studies heterogeneity, we provided a stratification outlining the use of AI in preoperative planning, intraoperative AI- assistance, real-time decision-making, surgical workflow optimization, and postoperative care/follow-up (Table 1).

**Table 1:** Evidence mapping on AI applications in management of benign esophageal disease

Evidence Domain	Clinical Functions	AI Technologies	Mapping Summary	Evidence Level
Precision Surgical Planning [18–39]	1. Anatomy segmentation +3D modeling for paraesophageal hernia planning 2. Quantification of diaphragmatic defects and esophageal length 3. Integration of manometry/pH-impedance/endoscopy for functional phenotyping 4. Predictive models for individualized perioperative risk	Machine learning, deep learning, automated 3-D reconstruction, predictive modeling	AI improves anatomical visualization, enhances surgical planning accuracy, and supports patient-specific risk stratification.	Mostly retrospective ML analyses No large prospective validations yet
Intelligent Intraoperative Assistance & Surgical Workflow Optimization [40–52]	1. Real-time identification of vagus nerve, crura, esophageal adventitia, vessels 2. Augmented reality overlays to guide dissection 3. AI-assisted fundoplication calibration to avoid tight wraps 4. Tremor reduction, motion scaling, fatigue compensation on robotic platforms 5. Intraoperative fusion of functional + anatomical data	Computer vision, convolutional neural networks (CNNs), augmented reality (AR), robotic AI modules	AI enhances precision and structure recognition, reduces variability, and supports functional surgical decision-making	Pilot CV/AR studies Early integration into robotic learning modules Limited prospective clinical evaluation
	1.OR time prediction and resource allocation 2.Real-time phase recognition in surgical videos 3.Postoperative surgeon performance metrics (motion efficiency, instrument use) 4.Support for continuous quality improvement and education	ML prediction models, automated video analysis, performance analytics	AI increases workflow efficiency and strengthens quality assurance across surgical teams	Early translation into surgical training Limited routine clinical implementation
AI-Enhanced Perioperative Management & Quality Optimization [53–55]	1. Early detection of leak, infection, stricture 2. Model-based prediction of postoperative complications 3. Telemonitoring + wearable-driven personalized rehabilitation 4. Dynamic adjustment of nutrition and recovery plans	Predictive analytics, continuous monitoring algorithms, wearable-sensor data integration	AI supports early complication recognition and personalized recovery, improving functional outcomes after benign esophageal surgery.	Few esophageal-specific datasets Strong potential but limited validation

### Preoperative planning and risk stratification

ML algorithms are increasingly being integrated into the management of esophageal benign disease [18]. Currently, AI enhances diagnostic accuracy by facilitating the analysis of endoscopic and radiologic studies, high-resolution esophageal manometry, and Functional Lumen Imaging Probe imaging [19, 20]. For instance, ML models can identify nuanced motility patterns in achalasia that may be difficult for human observers to discern, thereby enabling earlier and more accurate disease classification [21, 22]. In the assessment of dysphagia, AI algorithms evaluate swallowing function through video fluoroscopy, providing quantitative data that support informed clinical decision-making [23–25]. In a similar manner, AI-driven image analysis could in the near future enhance the visualization and characterization of Zenker diverticulum. Also, advanced algorithms can facilitate early identification of dysplastic and neoplastic lesions during surveillance endoscopy of Barrett’s esophagus, potentially allowing for timely intervention [26–28]. Beyond diagnostics, AI is contributing to the advancement of personalized treatment strategies. ML models can predict individual patient responses to interventions such as pneumatic dilation or peroral endoscopic myotomy (POEM) in achalasia, potentially optimizing therapeutic outcomes [29].

In the setting of HH and GERD, ML algorithms are capable of processing extensive datasets derived from endoscopy, radiology, high-resolution manometry, and pH-impedance tests, facilitating a more detailed characterization of the preoperative anatomical and functional variables [30–34]. This comprehensive insight enables surgeons to devise tailored surgical strategies, which is essential for optimizing patient outcomes and reducing postoperative recurrence rates. ML analyze large datasets of preoperative clinical variables (age, comorbidities, laboratory values) and historical surgical outcomes to provide individualized risk stratification for postoperative complications [35–37]. In benign esophageal surgery, this capability might be important to identify patients who may benefit from intensified perioperative optimization or alternative therapies. Predictive AI models contribute to shared decision-making by quantifying individualized surgical risk, enabling informed consent discussions and surgical candidacy evaluations with enhanced precision. Moreover, advanced AI algorithms facilitate automated three-dimensional reconstruction and segmentation of upper gastrointestinal anatomy from preoperative imaging modalities, enabling surgeons to visualize complex anatomical relationships and variations with greater precision. In procedures such as repair of paraesophageal hernia, AI-assisted imaging supports the quantification of hernia axial length and size, esophageal length, and size of diaphragmatic defects, thereby enhancing the accuracy of surgical planning [33]. This capability might be particularly relevant for preoperative preparation in cases involving diaphragmatic repair with mesh where 3-D printing may also provide a more precise surgical procedure and reduce operative time [38, 39]. Overall, AI and ML hold a great potential to transform the diagnosis, treatment, and long-term management of esophageal benign diseases, improving patient outcomes through tailored and data-driven approaches.

### Intraoperative AI assistance

The impact of AI is most tangible intraoperatively, augmenting surgical precision, safety, and efficiency [40]. A significant advancement in esophageal surgery might be the application of AI for real-time identification of critical structures through computer vision analysis of surgical video feeds as previously described for laparoscopic cholecystectomy [41–44]. Systems utilizing convolutional neural networks are capable of detecting and marking key anatomical features to be recognized during laparoscopic or robotic fundoplication. These include the vagus nerves, diaphragmatic crura, esophageal wall and vascular supply to the gastric fundus. The integration of augmented reality overlays provides intraoperative guidance to surgeons, facilitating the preservation of essential structures, minimizing the risk of unintentional injury, and improving clinical outcomes. This approach is particularly important in benign esophageal disease where preservation of vagal innervation and mucosal layer integrity is an important determinant of surgical quality.

Intraoperative AI applications are increasingly recognized as valuable assets in the management of Zenker diverticulum, achalasia, and Barrett esophagus. For Zenker diverticulum, AI and transoral septotomy systems have the potential to improve septum division precision, thereby minimizing procedural risks and enhancing patient outcomes [45, 46]. In the context of achalasia, AI supports real-time monitoring during POEM, facilitating muscle layer identification and reducing the likelihood of complications. Additionally, ML integration with indocyanine green may enhance mucosal visualization, potentially decreasing inadvertent mucosal injury during minimally invasive Heller myotomy [47]. Regarding Barrett esophagus, AI-powered imaging technologies assist clinicians in identifying dysplastic regions during endoscopic mucosal resection or radiofrequency ablation, supporting comprehensive and targeted treatment delivery [26–28]. During antireflux surgery, AI may prove beneficial in fashioning the fundoplication, thereby avoiding excessively tight wraps that could cause postoperative dysphagia. These systems offer the potential to reduce technical variability among surgeons. Incorporating intraoperative data with preoperative functional information obtained from high-resolution manometry and pH-impedance testing may inform decision-making regarding the optimal type of fundoplication and crural reconstruction. This approach can help optimize functional outcomes and reduce complications such as postoperative dysphagia or gas bloat symptoms. Overall, these AI-driven technologies hold promise for safer, more effective, and personalized intraoperative management of benign esophageal disorders.

The integration of AI algorithms within robotic surgery platforms is revolutionizing this field of surgery by enhancing instrument control, filtering tremor, optimizing hand motion scaling, and compensating for surgeon fatigue, thereby permitting precise dissection especially during revisional procedures [48–50]. Furthermore, AI-based learning modules embedded in robotic systems can recommend optimal instrument positioning, suture placement, and tension adjustment during complex surgical repairs such as crural closure in large HH. This may contribute to standardize surgical quality and reduce operative time.

### Surgical workflow optimization

In addition to direct procedural improvements, AI technologies have the potential to enhance the overall surgical workflow. For example, operative time prediction utilizes ML models to estimate procedure duration based on patient complexity and type of surgery, thereby supporting more effective operating room scheduling and resource management. Intraoperative video analysis involves AI-driven systems that automatically identify and annotate key surgical stages and critical events in real time, which contributes to retrospective quality improvement initiatives and advances in surgical education [51, 52]. Furthermore, surgeon performance feedback is facilitated postoperatively through AI-generated analytics that offer detailed insights into motion efficiency, instrument utilization, and adherence to procedural best practices, promoting ongoing professional development.

### Postoperative management and follow-up

Optimal recovery from surgical treatment of HH and GERD depends on early detection of complications and personalized rehabilitation strategies. AI models might analyze continuous postoperative clinical data streams (vital signs, lab results, symptom reporting) to detect early signs of complications such as leaks before clinical deterioration occurs [53]. In benign esophageal surgery, recognizing leaks or strictures at an earlier stage allows prompt conservative or endoscopic management and may reduce morbidity. Further, AI-driven analysis of patient-generated health data from wearable devices and telemonitoring platforms enables dynamic adjustment of postoperative care, nutritional plans, and rehabilitation protocols tailored to individual recovery trajectories [54, 55]. This personalized approach has the potential to improve functional outcomes and patient satisfaction.

#### Evidence mapping

**Fig. 2:**
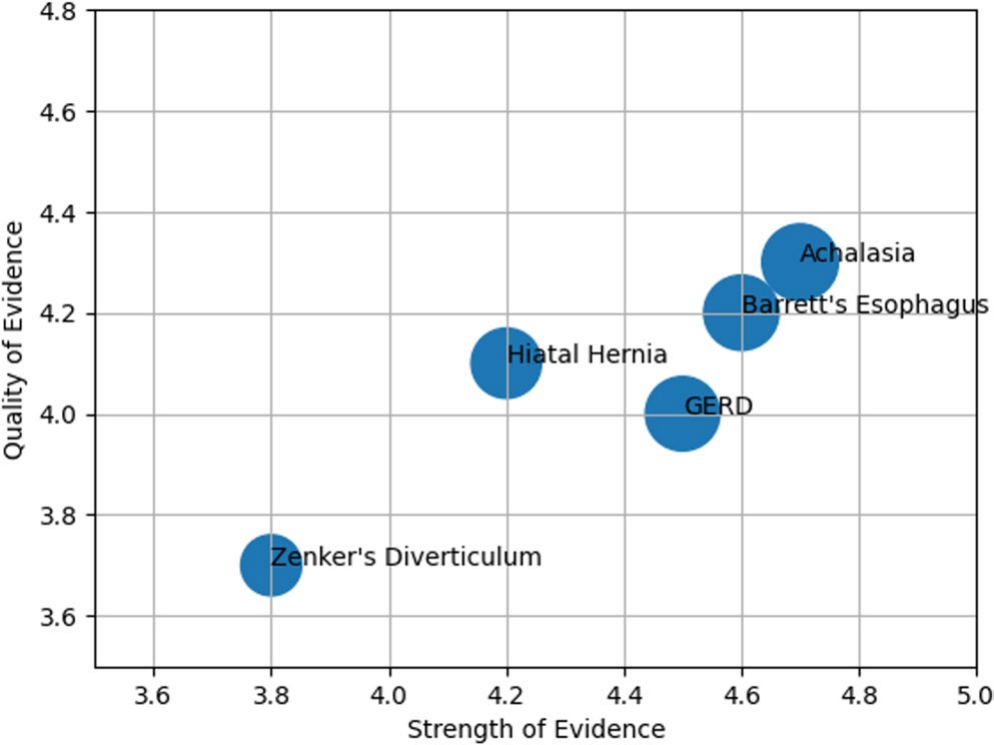
Evidence mapping stratified for the different application of AI in benign esophageal diseases. X-axis (strength of Evidence): the extent to which evidence supports the utility of AI (diagnosis, intraoperative use, outcomes). Y-axis (Quality of Evidence): overall methodological robustness (heterogeneity, clinical applicability). Bubble size: relative volume of available evidence

Across all stages of care, AI applications in benign esophageal surgery remain in early developmental phases, and published studies are still at an exploratory stage, with considerable room for improvement in terms of evidence quality, sample size and datasets, as well as research methods and technologies. Currently, evidence mapping shows promising benefits in Precision Surgical Planning, Intelligent Intraoperative Assistance, and AI-Enhanced Perioperative Management and Quality Optimization, but larger prospective and procedure-specific datasets are required to support widespread clinical integration. A comprehensive overview of the evidence mapping (Fig. 2) for AI applications in achalasia, Barrett’s esophagus, GERD, hiatus hernia, and Zenker diverticulum is presented in Table 2. We evaluate the AI application within the Technology Readiness Levels (TRL) framework to deliver an assessment focused on clinical applicability (https://www.esa.int/Enabling_Support/Space_Engineering_Technology/Shaping_the_Future/Technology_Readiness_Levels_TRL.) . This approach underscores the distinction between algorithmic results in controlled environments and practical readiness for real-world implementation, thereby elucidating translational relevance and potential AI-based implications for patient care.

**Table 2:** Evidence derived from the included studies, summarizing the strength and quality for AI applications across the considered benign esophageal conditions. Overall evidence strength reflects the extent and consistency of reported clinical utility across diagnostic, intraoperative, and postoperative domains. Evidence quality refers to the methodological robustness, clinical applicability, and consistency of findings. Key AI applications highlight the principal areas of implementation described in the literature, while the interpretation column provides an integrated assessment of the maturity and clinical relevance of AI for each condition. Technology Readiness Level (TRL)

Condition	Overall Evidence Strength	Overall Evidence Quality	Key AI Applications	Interpretation	TRL
Achalasia	Moderate	Moderate	Diagnosis (HRM), outcome prediction (POEM), intraoperative guidance	Moderate evidence across diagnostic and therapeutic phases	5-6
Barrett’s esophagus	Moderate	Moderate	Dysplasia detection, endoscopic surveillance, targeted therapy guidance	Moderate evidence in diagnostic endoscopy	5-6
GERD	Moderate	Low	Preoperative planning, risk stratification, intra/postoperative management	Heterogeneous applications across the care pathway	5–6
Hiatal hernia	Moderate	Low	Imaging analysis, surgical planning, anatomical reconstruction, risk prediction	Possible role in surgical planning and anatomical assessment	5–6
Zenker’s diverticulum	Low	Low	Intraoperative assistance (septotomy), imaging enhancement	Emerging field with promising but limited and less standardized evidence	3–4

## Discussion

AI is having a profound impact on the surgical management of benign esophageal disease. The main potential benefits of AI lay in improving diagnostic precision and optimizing surgical planning. ML algorithms can analyze large datasets from imaging techniques such as endoscopy, radiology, high-resolution manometry, pH-impedance, and Functional Lumen Imaging Probe, thereby enabling a comprehensive assessment of the anatomical and functional foregut abnormalities [56]. This detailed analysis might support surgeons in developing individualized surgical strategies, which is essential for optimizing patient outcomes and minimizing recurrence rates [57]. During intraoperative procedures, AI-powered systems are increasingly providing real-time support through advanced imaging modalities, augmented reality applications, and robotic platforms. These technologies improve visualization of the surgical field and critical anatomical landmarks, potentially contributing to reduced complication rates, enhanced identification of structures, and improved assessment of crural closure and fundoplication appropriateness. Moreover, AI demonstrates considerable value in postoperative care through elaboration of predictive models that can evaluate patient-specific risks, such as the likelihood of surgical failure or complications. This capability may facilitate preemptive management strategies. AI-powered analytics also support the ongoing monitoring of patient outcomes and long-term follow-up, contributing to evidence-based enhancements in surgical practice. Nonetheless, significant challenges remain, including the need for validation studies, ethical considerations surrounding data privacy, and the integration of AI tools into established clinical workflows [58–60]. As AI continues to evolve, its implementation in the surgical treatment of benign esophageal disease holds substantial potential to elevate standards of care by fostering safer, more effective, and personalized approaches tailored to each patient’s unique condition.

Most AI applications in upper gastrointestinal surgery currently stem from cancer-focused surgical datasets. To support the rigorous development and validation of AI models specific to benign esophageal disease, it is essential to build dedicated, large-scale and multicenter datasets. Key ethical imperatives include maintaining strict patient confidentiality, ensuring transparency of algorithms, and preserving surgeons’ autonomy [61, 62]. To ensure transparency it may be worth investigating also the potential use of deductive AI methodologies, like those based on Answer Set Programming (ASP) [63, 64]. By encoding clinical knowledge into ASP rules, healthcare providers can ensure that their decisions consistently meet ethical and safety standards and support the informed consent process.

Furthermore, successful adoption of AI systems requires user-friendly interfaces and structured training initiatives to ensure seamless integration into routine surgical practice. Future advancements should aim to address unmet clinical needs through innovations such as AI-driven surgical training platforms for early-career foregut surgeons including automated video editing, standardized video archives, and sophisticated simulation tools capable of providing real-time feedback and analysis of instrument’s trajectories and operator’s hand motion [65–67]. Additionally, deep learning–based intraoperative video analysis offers opportunities to implement continuous quality assurance, promote standardization of functional foregut procedures, and minimize inter-operator variability. Ultimately, the development of AI-enabled, multimodal decision-support systems that synthesize preoperative imaging, intraoperative metrics, and comprehensive biological data from genomics to microbiome profiles holds promise for advancing personalized surgical care and optimizing treatment outcomes.

Artificial intelligence has advanced beyond theoretical or exploratory uses and is now generating clinically actionable outcomes in the perioperative management of benign esophageal disease [68]. Nonetheless, widespread implementation is limited by the scarcity of high-quality, disease-specific datasets and the necessity to ensure algorithmic robustness, generalizability, and bias mitigation. Successful integration will require seamless incorporation into current clinical workflows, prioritization of user-centered design, and specialized training initiatives. Hybrid and interpretable AI solutions may further accelerate clinical adoption. In summary, artificial intelligence has matured sufficiently to influence crucial aspects of benign esophageal surgery, though widespread adoption will depend on coordinated validation, standardization, and regulatory efforts.

### Limitations

This scoping review has several limitations. Despite the use of a comprehensive search strategy, relevant studies may have been inadvertently missed or excluded during the screening process; to minimize this risk, screening was conducted independently by three reviewers. Publication bias represents a key limitation, particularly given the predominance of retrospective studies and case series among the included studies thereby reducing the overall strength of the evidence. Additionally, substantial heterogeneity in study design, populations, and outcome measures limited the comparability of findings and precluded meaningful synthesis. The absence of a meta-analysis prevented quantitative pooling of data and restricted the precision of effect size estimation. Finally, restricting inclusion to English-language publications may have introduced bias, potentially excluding relevant studies and affecting the comprehensiveness of the review.

## Conclusion

AI has the potential to impact the entire surgical care continuum for the surgical treatment of benign esophageal disease by enhancing diagnostic accuracy, preoperative planning, intraoperative precision, and postoperative management. The utilization of AI algorithms might also facilitate standardized education and training for young foregut surgeons and reduce learning curves of both laparoscopic and robotic surgical procedures. The full benefits of AI in surgery for benign esophageal disease will depend on the implementation of robust research, responsible ethical integration, and comprehensive surgeon education.

## Data Availability

No datasets were generated or analysed during the current study.
